# Angiogenesis and chemotherapy resistance: optimizing chemotherapy scheduling using mathematical modeling

**DOI:** 10.1007/s00432-021-03657-9

**Published:** 2021-05-29

**Authors:** Mariusz Bodzioch, Piotr Bajger, Urszula Foryś

**Affiliations:** 1 Faculty of Mathematics and Computer Science, University of Warmia and Mazury in Olsztyn, Sloneczna 54, 10-710 Olsztyn, Poland; 2 Faculty of Mathematics, Informatics and Mechanics, University of Warsaw, Banacha 2, 02-097 Warsaw, Poland

**Keywords:** Tumor growth, Angiogenesis, Anti-angiogenic treatment, Chemotherapy, Resistance, Optimal control, Mathematical modeling, 49K15, 92C50, 37N25

## Abstract

Chemotherapy remains a widely used cancer treatment. Acquired drug resistance may greatly reduce the efficacy of treatment and means to overcome it are a topic of active discussion among researchers. One of the proposed solutions is to shift the therapeutic paradigm from complete eradication of cancer to maintenance, i.e., to treat it as a chronic disease. A concept of metronomic therapy (low chemotherapy doses applied continuously) emerged in early 2000s and was henceforth shown to offer a number of benefits, including targeting endothelial cells and reducing acquired drug resistance. Using mathematical modeling and optimal control techniques, we investigate the hypothesis that lower doses of chemotherapy are beneficial for patients. Our analysis of a mathematical model of tumor growth under angiogenic signaling proposed by Hahnfeldt et al. adapted to heterogeneous tumors treated by combined anti-angiogenic agent and chemotherapy offers insights into the effects of metronomic therapy. Firstly, assuming constant long-term drug delivery, the model suggests that the longest survival time is achieved for intermediate drug doses. Secondly, by formalizing the notion of the therapeutic target being maintenance rather than eradication, we show that in the short term, optimal chemotherapy scheduling consists mainly of a drug applied at a low dose. In conclusion, we suggest that metronomic therapy is an attractive alternative to maximum tolerated dose therapies to be investigated in experimental settings and clinical trials.

## Introduction

In this study, we introduce a mathematical model of tumor growth under angiogenic signaling and discuss how it can be used to support clinicians in choosing the right chemotherapy scheduling protocols.

Chemotherapy remains one of the treatment strategies most frequently used to combat cancer. As chemotherapy is not selective, it affects both the tumor and the host’s healthy cells alike and hence requires careful dosing so as not to induce too severe side-effects. Therefore the establishment of the “Maximum Tolerated Dose” (MTD) for a given drug is one of the aims of phase I clinical trials. Once MTD is established, the treatment typically consists of administering an MTD drug dose to kill as many cancer cells as possible, followed by a prolonged drug-free interval to allow the host enough time to recover. The main issue associated with this approach is that genetic instability of tumor cells, coupled with their high proliferation rate, often lead to development of resistance to chemotherapy. As a result, even though the initial response to treatment may be promising, subsequent chemotherapy cycles become less and less effective.

It has therefore been proposed that it may be beneficial to administer a drug at a lower density, but continuously (the so-called metronomic scheduling), thus treating cancer as a chronic disease and shifting the therapeutic paradigm from total eradication to maintenance (Scharovsky et al. 2009; Fidler and Ellis 2000; Afrasiabi et al. 2020). Metronomic therapy has been reported to have a number of benefits: immune system boosting, inducing anti-angiogenic effects (Hanahan et al. 2000; Pasquier et al. 2010), and preventing acquired drug resistance (Kareva et al. 2015). In this study, we focus on the latter two aspects of metronomic, low-dose therapies. In particular we use mathematical modeling to provide insights into the following hypothesis: appropriate chemotherapy dosing could prolong patient survival by maintaining tumor size at lower, non-life-threatening levels. Furthermore, this is achieved by targeting blood vessels and delaying the onset of drug resistance.

The drug dosing is therefore essential to successful treatment. Given the quantitative nature of this issue, it seems intuitive that mathematical techniques could be employed to support clinicians in their decision-making process (Gatenby and Maini 2003; Michor and Beal 2015). The main motivation behind modeling is that an experimental oncologist, equipped with an appropriate mathematical model, could run computer simulations to preliminary assess different treatment protocols. This process, combined with experience and intuition of the researcher, could lead to an identification of the most promising treatment schemes to be then tested experimentally. Similarly, a clinician having to decide which chemotherapeutic schedule to use to treat a patient could make a better-informed decision based on additional inputs from mathematical simulations which predict tumor responses to different types of treatment. If a model was particularly well supported by experiments and had enough data, it could even be used to propose optimal treatment schedules at an individual level. Hence, our work fits into the general trend of usage of mathematical modeling as a tool in personalized therapies: see, e.g., Komarova and Boland (2013); Agur et al. (2020), or Ottesen et al. (2020) for a more mechanistic approach.

Mathematical models of tumor growth have existed in the literature for a long time. Historically, notable examples include the early works by Laird (1964) in the 1960s where a simple, Gompertzian model was fitted to experimental data from mouse, rat, and rabbit tumors. In 1999, Hahnfeldt et al. (1999) developed a now classic model of tumor angiogenesis, adapted in many other studies (this one being no exception). More recently, noteworthy are the works by Gatenby et al. (2009) on adaptive therapy schedules which employed mathematical modeling techniques.

The mathematical framework we employ in this study to numerically compute optimal treatment is optimal control theory. The very first question which needs addressing is what do we mean by “optimal”. Historically mathematicians attempted to minimize the objective functional which is a sum of overall tumor burden, which can be thought as an area below a curve describing the tumor cell density (or, in case of compartmental models, weighted density across compartments with the resistant component receiving a higher penalty), and the tumor size at the end of the treatment (Ledzewicz and Schättler 2014, 2014, 2002; Świerniak et al. 2016; Świerniak and Śmieja 2001; Śmieja and Świerniak 2003). Usually, an additional penalty on overall administered drug amount is imposed to penalize toxic side-effects. While these methods have proved themselves to be quite successful and leading to important insights, we take a slightly different approach. As the goal of metronomic therapy is to shift the therapeutic paradigm from maximizing cell kill to maintaining the tumor at a non-life-threatening level, we decided to include an additional term in our objective functional which adds a running penalty for periods of time in which the tumor is in a drug-resistant state (i.e., there is more resistant than sensitive cells). We do this in effort to see if penalizing drug resistance may lead to better therapeutic outcomes in terms of long-term patient survival. Note that in this study we assume that we are treating a tumor whose intrinsic properties (drug resistance) make it incurable using cytotoxic agents, as is sometimes the case (Savage et al. 2009). The goal is therefore to prolong patient survival, rather than to actually cure the tumor.

In this study we use a mathematical model which tracks: the number of chemotherapy-sensitive tumor cells and the number of chemotherapy-resistant tumor cells, as well as a variable carrying capacity which is related to the size of the vasculature. Using a simpler model with just two tumor subpopulations and constant carrying capacity we have already shown that smaller doses of chemotherapy could indeed yield better survival times for patients (Bajger et al. 2019). Here, we incorporate the ideas of Hahnfeldt et al. (1999) to be able to include the anti-angiogenic effects of metronomic therapy, as well as the potential effects of anti-angiogenic treatment.

The original Hahnfeldt’s model was validated using experimental data from mice under anti-angiogenic treatment. Hahnfeldt and collegues assumed that the evolution of a vascular network that supplies nutrients and oxygen to tumor cells strictly controls the tumor growth. They introduced the concept of carrying capacity of the vasculature and provided a framework to account the effects of anti-angiogenic therapies. The size of the vasculature controls the limiting size of the tumor, while the growth of the vasculature is controlled by the tumor cells secreting pro- and anti-angiogenic factors.

Despite the initial high hopes associated with anti-angiogenic treatment, it became apparent that anti-angiogenic therapy does not yield a significant improvement in long-term patient survival (Jain 2005). We therefore consider it combined with more classical, chemotherapeutic treatment, although we take a rather simplistic approach. In general, the interplay between the two therapies is quite subtle. Contrary to our intuition, anti-angiogenic agents may have a “normalizing” effect on abnormal tumor vasculature, thus leading to an improved flow and hence improved chemotherapy delivery mechanism (Goel et al. 2012). To make the best use of this mechanism a careful dosing schedule has to be chosen. We do not model this phenomenon explicitly in this study and focus more on acquired drug resistance (ADR), while noting that this is a possible extension of our model. For works on mathematical modeling in which vessel normalization is taken into account, we refer the reader to Poleszczuk and Skrzypczak (2011) and Alarcón et al. (2006).

To model ADR, we subdivide the population of tumor cells into two subpopulations: one sensitive to chemotherapy and the other completely resistant. In addition, we include a flow between the two cellular compartments due to genetic mutations which may be important in particular for long-time horizon analysis. This extended model can be used to model both the effects of chemotherapy (and how drug resistance emerges) and the anti-angiogenic treatment. To gain some initial intuition about the behavior of the system, we first consider its behavior when the chemotherapy dose is constant in time. This is meant to be an approximation of a continuous, indefinite long-term chemotherapeutic treatment. We then consider an optimal control problem, i.e., we allow the chemotherapeutic drug dose to vary over time. Finally, we consider more clinically realistic approximations to the optimal controls and show that they give near-optimal results.

The paper is organized as follows: in Sect. 3, we present the main conclusions of our study obtained using numerical simulations of our model. In Sect. 3.1, as a first and simplest approximation of an actual therapeutic protocol, we consider anti-angiogenic and chemotherapy doses as constant in time and optimize patient survival time. It gives us basic insights into the dynamics of the considered model. In Sect. 3.2, we consider 14-day therapeutic protocols that minimizes the tumor volume and penalizes drug resistance. We use optimal control framework to numerically compute optimal treatment protocols. It may have significant impact on the optimal or suboptimal solution. In Sect. 4, we discuss the obtained results.

The remaining sections are devoted to a more theoretical description of the model. In Sect. 2, we list the assumptions under which the model described in Sect. 2.1 was build and list the nominal parameter values. In 2.2, we formally introduce our objective functional constructed to target the problem of drug resistance. In Sect. 2.3, we describe the numerical methods that were used throughout the paper. Finally, Sect. 5 contains conclusions.

Formal, mathematical analysis of the model is confined to the Appendices. In Appendix A.1, we formulate basic properties of the mathematical model, and in Sect. A.2, we investigate the existence of stationary states and formulate conditions for their stability.

## Methods

In this section, we describe a mathematical model which we used during our study and numerical methods allowing for illustrate presented results. Below we list the assumptions under which the model described in Sect. 2.1 was built.

1. Tumor cell population is subdivided into two subpopulations: drug-sensitive and drug-resistant. We have analyzed subdividing the population into more compartments with gradually increasing resistance and concluded that the benefit is small relative to an increase in model complexity.
2. Each tumor subpopulation, in the absence of the other one, follows the Gompertz growth model. See discussion in Bodnar and Foryś (2007) on other growth models (e.g., logistic).
3. Carrying capacity of the environment is tied to the availability of the vasculature. The process of angiogenesis (i.e., new blood vessels sprouting) is modeled according to the equations proposed by Hahnfeldt et al. (1999).
4. It is assumed that a constant dose of anti-angiogenic agent is supplied throughout the treatment. Rather than optimizing for both chemotherapy and the anti-angiogenic drug dose, we focus on a slightly different question: Given an anti-angiogenic agent constant dose, what is the optimal chemotherapy schedule?
5. Chemotherapy-sensitive cells are killed by the drug according to the log-kill hypothesis, i.e., a death rate is proportional to the number of cells and the concentration of the drug. It is assumed that the drug has no effect on the resistant subpopulation, and that we can control the drug concentration at all times. Drug metabolism is ignored for simplicity.
6. The flow between resistant and sensitive compartments is modeled by assuming constant mutation rates proportional to cell densities. This assumption is motivated by works of Luria and Delbrück (1943). For a detailed discussion of different modeling approaches, see Foo and Michor (2014).

### Mathematical model formulation

In the original Hahnfeldt et al.’s model (Hahnfeldt et al. 1999), two variables have been introduced. The first one, *V*, reflects the tumor volume, while the second one, *K*, is the “carrying capacity”, which is related to the size of the vasculature that can supply tumors of that volume. Hahnfeldt et al. assumed that the tumor population follows the Gompertzian-type of growth. Thus, the tumor dynamics is described by the equation

$$\begin{aligned}\dot{V} = -\lambda V \ln \frac{V}{K}\end{aligned}$$

where $\lambda$ reflects maximal growth rate of the tumor.

On the basis of the hypothesis that the tumor growth is precisely dependent on the development of the vasculature, the fixed carrying capacity of the Gompertzian growth was replaced by the time-dependent variable *K*(*t*). Note that the tumor growth described by the above equation will be bounded only if the tumor vasculature tends to a limited maximum, i.e., if *K*(*t*) were exponentially growing, *V*(*t*) would exhibit an asymptotic exponential growth with the same constant rate. Developing the equation for *K*, Hahnfeldt and colleges have taken into account both stimulatory and inhibitory factors, as well as natural loss rate of vessels and the possible anti-angiogenic action of administrated drugs. Thus, the equation for *K*, which includes those four factors is

$$\begin{aligned} {\dot{K}}=-\mu K + bV - d V^{2/3}K - \gamma Kv(t), \end{aligned}$$

where $\mu$ is the natural death rate of the endothelial cells, *b* is the rate at which the vasculature growth is stimulated by the cancer cells, *d* is a measure of how strongly the cancer cells inhibit the vasculature, and $\gamma$ is a sensitivity rate of the vasculature to the therapy with *v*(*t*) being the concentration of the drug. For the unperturbed growth, the negative term $d V^{2/3}K$ determines the saturating behavior of the tumor growth.

**Fig. 1:**
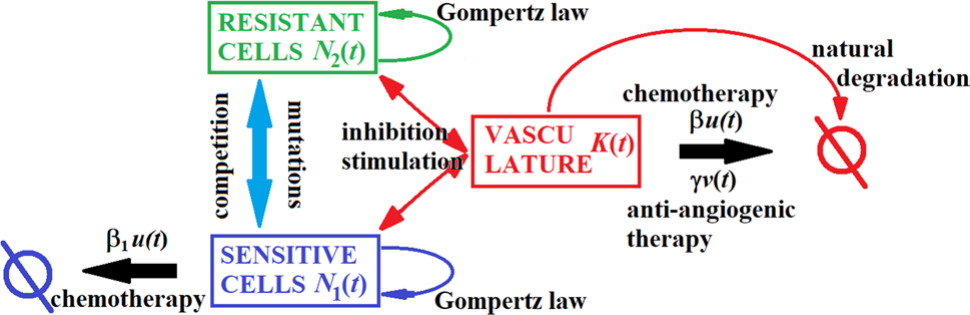
Scheme of a mathematical model described by Eq. (1)

The model proposed by Hahnfeldt et al. (1999) is derived assuming the tumor population is homogeneous. In this paper, we propose an extension of the Hahnfeldt et al.’s model encompassing chemotherapy resistance. Schematic representation of our model is presented in Fig. 1. We consider the tumor population as a heterogeneous one and subdivide the malignant cell population into two compartments that differ in their sensitivity to chemotherapy: sensitive ($N_1$) and resistant ($N_2$). As the underlying reason of ADR is due to genetic instability of cancer cells and errors occurring during replication (Gottesman 2002), constant mutation rates between the compartments are also included in the model. The effect of the chemotherapeutic drug on the sensitive cells is proportional to the potency of the chemotherapeutic, the concentration of the drug at time *t* and the number of cells, and hence, the model is in accordance with the log-kill hypothesis (Skipper 1986). The above assumptions lead to the following system of differential equations:

1 $$\begin{aligned} \begin{aligned} {\dot{N}}_1&= -\lambda _1 N_1 \ln \frac{N_1+N_2}{K} - \tau _1 N_1 + \tau _2 N_2 - \beta _1 N_1 u(t), \\ {\dot{N}}_2&= -\lambda _2 N_2 \ln \frac{N_1+N_2}{K} + \tau _1 N_1 - \tau _2 N_2, \\ {\dot{K}}&= -\mu K + b \big (N_1 + N_2\big ) - d \big (N_1 + N_2 \big )^{2/3}K - \beta Ku(t)-\gamma K v(t). \end{aligned} \end{aligned}$$

Here: *u*(*t*) and *v*(*t*) are the chemotherapy and anti-angiogenic treatment doses at time *t*, respectively; $\tau _1$ is the rate of mutations of sensitive cells to resistance and $\tau _2$ is the rate of back mutation from resistance to sensitivity; $\beta$ and $\gamma$ are sensitivity rates of the vasculature to the chemotherapeutic and anti-angiogenic agents, respectively. We assumed that the mutation affects only drug resistance. It means that there is no difference between secretion rates of pro-angiogenic and anti-angiogenic factors of both types of cancer cells. Details on the derivation of the equation for *K* and descriptions of the remaining parameters can be found in the original article (Hahnfeldt et al. 1999). For a brief description of parameters’ roles and nominal values, see Table 1.

Let us denote the initial tumor size at time $t=0$ by $N_\mathrm{{init}}$ and by $N_\mathrm{{sat}}$ the maximal (saturating) tumor size potentially sustainable by the vasculature. The doubling time *T* can be calculated using the formula

$$\begin{aligned} T = \frac{\ln 2}{\lambda \left( \ln N_\mathrm{{sat}} - \ln N_\mathrm{{init}}\right) }, \end{aligned}$$

where $\lambda$ is the population growth rate. Note that the denominator in the expression above is defined as the instantaneous growth rate of the tumor at the start of the simulation. Initiating the simulations with $N_1(0)=280\ \text {mm}^3$, $N_2(0)=20\ \text {mm}^3$ and $K(0)=650\ \text {mm}^3$ and taking $N_\mathrm{{sat}}=17{,}000\ \text {mm}^3$, we can calculate numerically the doubling time as 31 h.

**Table 1:** Nominal parameter values

Name	Value		Unit	Role	Reference
$\lambda _1$	1.92	$\times 10^{-1}$	1/day	Proliferation rate of sensitive cells	Hahnfeldt et al. (1999)
$\lambda _2$	0.96	$\times 10^{-1}$	1/day	Proliferation rate of resistant cells	
$^* \tau _1$	2.00	$\times 10^{-5}$	1/day	Mutation rate toward the resistant phenotype	
$^* \tau _2$	1.00	$\times 10^{-5}$	1/day	Mutation rate toward the sensitive phenotype	
$\mu$	0.00		1/day	Natural death rate of endothelial cells	Hahnfeldt et al. (1999)
*b*	5.85		1/day	Vascular growth rate stimulated by cancer cells	Hahnfeldt et al. (1999)
*d*	8.73	$\times 10^{-3}$	day$^{-1}\,$vol^-2/3^	Vascular inhibition rate by cancer cells	Hahnfeldt et al. (1999)
$^*\beta _1$	0.3		day$^{-1}\,$conc^-1^	Sensitivity rate of sensitive cells to the chemotherapy agent	
$^*\beta$	0.1		day$^{-1}\,$conc^-1^	Sensitivity rate of the vasculature to the chemotherapy agent	
$\gamma$	2		day$^{-1}\,$conc^-1^	Sensitivity rate of the vasculature to the anti-angiogenic agent	
*u*(*t*)	[0, 1]			Concentration of chemotherapy	
*v*(*t*)	[0, 1]			Concentration of anti-angiogenic agent	
$N_\mathrm{{init}}$	300		mm^3^	Initial volume of the tumor	
$N_\mathrm{{sat}}$	17,000		mm^3^	Saturating volume of the tumor in the absence of therapy	
$N_\mathrm{{crit}}$	8000		mm^3^	Critical (fatal) volume of the tumor	
$T_S$			day	Survival time	
$N_1$			mm^3^	Sensitive population	
$N_2$			mm^3^	Resistant population	
*K*			mm^3^	Carrying capacity	

Parameters marked with ^∗^ are varied between simulations (see text)

It should be noted that by non-dimensionalizing with respect to time, any change of the proliferation rate is equivalent to changing the units of time. Assuming drug administration duration is also scaled, the overall dynamics is otherwise unchanged. Hence, our conclusions can be applied for a family of values of the proliferation rate. For example, making a suitable rescaling of time, if we were use the 96 h doubling time of colorectal cancer (Rew and Wilson 2000), we would get the reduced value of $\lambda _1$ and the conclusion is unchanged. Similarly, making an appropriate scaling of the initial condition or fatal volume of the tumor, our conclusions hold for a family of initial or fatal tumor volumes.

**Fig. 2:**
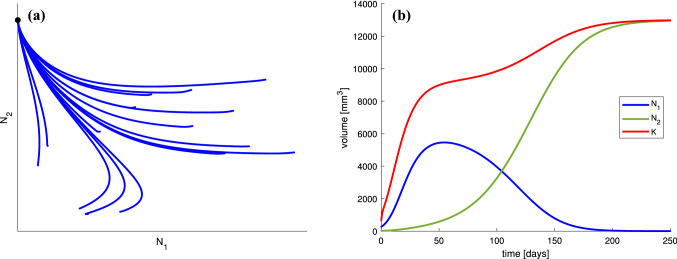
**a** Phase portrait and **b** numerical solution for system (1). The chemotherapy dose is $u=0.25$ and the anti-angiogenic agent dose is $v=0.5$. In **b**, the initial condition is chosen as [280, 20, 650]

Mathematical properties of the model described by Eq. (1) are presented in Appendix. Here, we only present exemplary dynamics of the model under the assumption that both drugs are applied with constant rates. In should be noted that for biologically relevant values of *u* and *v*, the system always has a positive steady state which is attractive. However, as we see in Fig. 2, this steady state represents resistant tumor; moreover, the tumor size at this state is far too large to be safe for the patient.

### Optimal control problem

In our earlier paper (Bajger et al. 2019), we have proposed an objective functional constructed to target the issue of drug resistance, which penalizes resistant populations. Comparing to objective functionals present in the literature, in our functional, apart from the standard terms which penalize the tumor size during the treatment and at the end of the treatment, a non-standard term penalizing time period during which the tumor is resistant is introduced. The following term

$$\begin{aligned} f(N_1,N_2) = \frac{\xi }{2}\left( 1+\tanh \left( \frac{N_2-N_1}{\epsilon }\right) \right) \end{aligned}$$

represents a general explicit running penalty for resistance. It may be thought of as a “smoothed-out” Heaviside-type function which jumps between 0 and $\xi$ depending on whether the majority of tumor cells are sensitive or resistant. The role of $\epsilon$ ($0<\epsilon \ll 1$) is to control the steepness of the slope.

Thus, the optimal control problem can be formulated as follows: find a measurable function $u:[0,T]\rightarrow [0,1]$ for a given fixed terminal time *T*, which minimizes the functional

$$\begin{aligned}&J(u(\cdot )) = \omega _1N_1(T) + \omega _2N_2(T) \\ &+ \int _0^T\left( N_1(t)+\eta _2 N_2(t)+\frac{\xi }{2}\left( 1 +\tanh \left( \frac{N_2(t)-N_1(t)}{\epsilon }\right) \right) +\theta u(t)\right) dt, \end{aligned}$$

under the dynamics of System (1). Here, all parameters $\omega _1$, $\omega _2$, $\eta _1$, $\eta _2$, $\xi$, $\theta$ are non-negative weights. The terms involving $\omega$ and $\eta$ penalize the size of the whole population at the end and during chemotherapy, respectively. The term $\theta u(t)$ is included to minimize side-effects of the chemotherapy.

**Table 2:** Nominal parameter values

Name	Value		Unit	Role
$\omega _1$	5.00			Weight for a final number of
				sensitive cells in the objective functional
$\omega _2$	2.50	$\times 10^1$		Weight for a final number of
				resistant cells in the objective functional
$\eta _1$	1.00			Weight for a running number of
				sensitive cells in the objective functional
$\eta _1$	5.00			Weight for a running number of
				resistant cells in the objective functional
$\xi$	1.00	$\times 10^3$		Weight for a resistance penalty in the
				objective functional
$\varepsilon$	1.00	$\times 10^1$		The “margin” parameter in the
				resistance penalty function
$\theta$	0.00		1/conc	Running drug dose penalty in the
				objective functional

It should be noted that for much simpler model analyzed by us in our earlier work we partially know the structure of optimal control (Bajger et al. 2019). We were able to prove that the optimal dosage ends with MTD. Moreover, we have shown numerically that for the simpler model the optimal control is of the form: MTD—intermediate dose—MTD. We therefore expect that an intermediate dosage will become part of the optimal solution in the more complex model analyzed in this study as well.

### Numerical methods

We choose the numerical approach “First Discretize then Optimize” to solve optimal control problem. To model the optimal control problem, a Python-based open-source optimization modeling language Pyomo with its algebraic equation extension Pyomo.DAE is used. We use the forward Euler method with regularly spaced grid of 400 points to discretize the problem. We have found that increasing the number of points beyond 400 has no effect on the solution. The discretization of the control problem on a fine grid leads to a large-scale non-linear programming problem. To find the optimal control numerically, we use underlying non-linear optimization algorithms like IPOPT, which is an implementation of primal-dual interior point method. IPOPT can be used to robustly solve constrained non-linear programming problems. It implements an interior point line search filter method to find a locally optimal solution for the problem. Choosing the error tolerance $10^{-12}$ in IPOPT, we can expect that the state variables are correct up to 7–9 decimal digits. To solve system (1) numerically, we use the standard MATLAB solver ode45 with error tolerance equal to $10^{-6}$.

## Results

We present results of our study performed on the basis of the mathematical model introduced and described in more details in Sect. 2. Our main goal is to extend survival time for patients by means of preventing drug resistance.

### Long-time horizon treatment

For the purpose of this section, we assume a theoretical scenario in which we are able to apply constant doses of both anti-angiogenic and chemotherapy for a long time in a continuous manner, that is, *u* (chemotherapy dose) and *v* (anti-angiogenic agent dose) are constant. Here, the drug doses are expressed as a fraction of MTD, so both *u* and *v* range between 0 and 1. This is of course oversimplification, but we would like to check which dose of chemotherapy leads to longest survival in such theoretical treatment scheme. As noted in Introduction, we define the survival time (denoted by $T_\mathrm{s}$) as the time needed for the tumor to reach the critical (fatal) tumor volume (denoted by $N_\mathrm{{crit}}$) which we assume to be the volume reflecting maximal possible size.

**Fig. 3:**
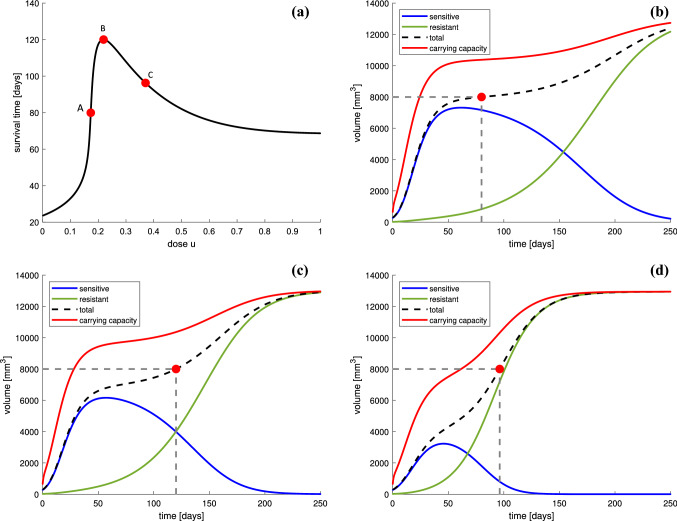
**a** Survival times for different chemotherapy doses in our model. Red point *B* depicts the maximum survival time; here, $T_s = 120$ days correspond to $u=0.2192$. Points *A* and *C* are chosen to represent different behaviors of the tumor cells population for $u=0.1732$ ($T_s = 80$ days) and $u=0.3704$ ($T_s=96$ days), respectively. **b**–**d** Evolution of the volumes of sensitive and resistant subpopulations, carrying capacity and total volume of the tumor for three different values of chemotherapy *u* corresponding to the points *A*, *B*, and *C* in **a**. The dotted lines mark the point at which the tumor volume reaches the critical level $N_\mathrm{{crit}}$. The initial condition is chosen as [280, 20, 650]

**Fig. 4:**
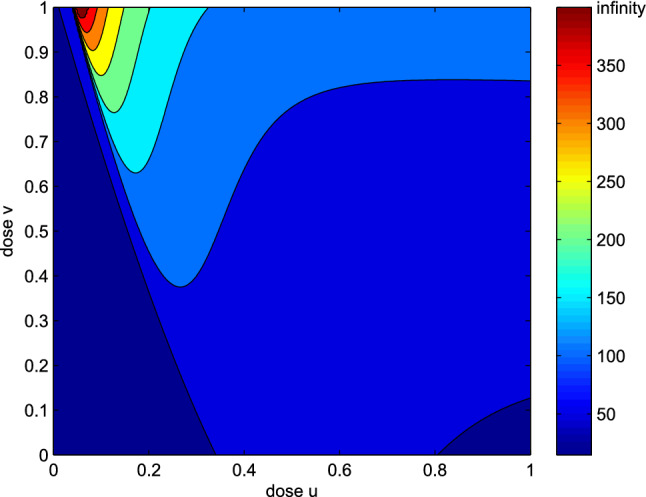
Dependence of the survival time on the chemotherapy and anti-angiogenic agent doses. The range “infinity” on the heatmap denotes such doses *u* and *v* for which the fatal volume $N_\mathrm{{crit}}$ is not reached. The initial condition is chosen as [280, 20, 650]

Figure 3 shows the survival time plotted against chemotherapy dose *u* and fixed anti-angiogenic agent dose $v=0.5$. Simulations were performed for different chemotherapy doses and the time needed for the tumor to reach a critical size $N_\mathrm{{crit}}$ was recorded in each case. Local maximum is clearly visible, which means that an intermediate (less than 25% of MTD) value of chemotherapy is predicted to be optimal and prolong the survival time the most. Plots 3b, 3c and 3d, respectively, show the cell population dynamics for three values of chemotherapy dose *u*, which are depicted by *A*, *B*, and *C* in Fig. 3a. Analysis for a similar model which did not include the process of angiogenesis was conducted in Monro and Gaffney (2009). In scenario *A*, where the chemotherapy dose is lower than the optimal, $u = 0.1732$, the tumor consists almost exclusively of sensitive cells. The chosen dose is too small to effectively inhibit the tumor growth and the survival time is shorter. In scenario *B*, the dose is enough to maintain the tumor volume below the fatal for 120 days. As it is expected, an increase in the chemotherapy dose promotes the growth of the resistant subpopulation. When the administrated dose is too large (here $u=0.3704$), a quick outburst of resistant subpopulation is present and it dominates over the sensitive one, which causes the survival time is shorter again. This observation supports the hypothesis (Scharovsky et al. 2009) that the longest survival time occurs when non-trivial competition between cellular subpopulations is present. It also suggests that there is a specific balance between killing sensitive cells by the drug and promoting the production of resistant cells that prolongs the survival time the most.

Figure 4 shows the dependence of the survival time on the chemotherapy and anti-angiogenic agent doses. It should be noted that for intermediate doses of chemotherapy, a small change in the anti-angiogenic agent dose may have a significant impact on the survival time. On the other hand, survival time is much less dependent on the anti-angiogenic agent dose when the chemotherapy dose is either very small, or very large. What is more, for particular values of *u* and *v*, the critical volume $N_\mathrm{{crit}}$ may not be reached. It means that for a specific combination of doses, in this theoretical scenario, the treatment is effective enough to maintain the tumor below the critical size indefinitely.

**Fig. 5:**
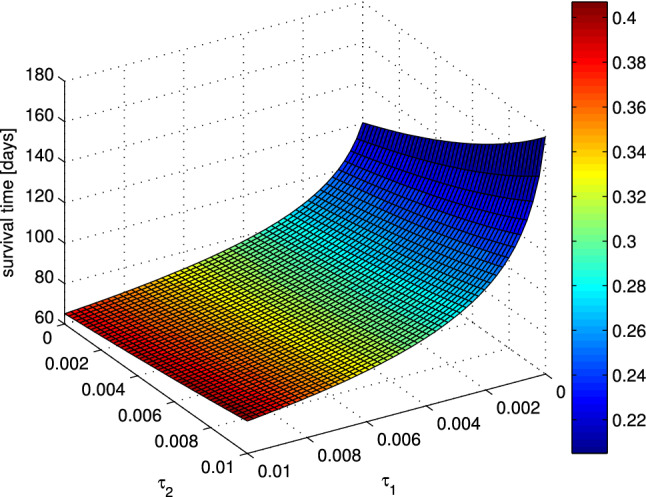
Dependence of the maximum survival time on the mutation rates $\tau _1,\tau _2\in \left[ 10^{-5},10^{-2}\right]$. Color represents the chemotherapy dose which yields the maximum survival time. The initial condition is chosen as [280, 20, 650] and the anti-angiogenic agent dose as $v = 0.5$

Although it is widely agreed that the emergence of ADR is due to mutations, the exact numerical values of the relevant parameters associated with this process are hard, if not impossible, to estimate. We denote by $\tau _1$ and $\tau _2$ the mutation rates from sensitivity to resistance and reverse, respectively. The rate of mutations per cell cycle has been estimated in Goldie and Coldman (1998) to be of the order of $O(10^{-7}-10^{-4})$. As the values of mutation rates strongly depend on the aggressiveness of the tumor, a set of simulations were performed for different values of mutation rates and the time needed for the tumor to reach a critical size together with the corresponding optimal dose was then recorded (see Fig. 5). Simulations were performed for mutation rates $\tau _1,\, \tau _2\in \left[ 10^{-5},10^{-2}\right]$. One can see that the smaller the value of $\tau _1$ (the mutation towards resistance) is, the longer survival time becomes and the lower dosage must be applied. The influence of the value $\tau _2$ (mutation from resistance) on the optimal dose is opposite, but less severe.

### Short-time horizon optimization

Minimizing tumor volume while imposing some constraints on the drug dosage are standard goals used in mathematical modeling. We postulate that to better reflect the aims of the “maintenance” therapeutic paradigm, we should also add a penalty for drug resistance. We will call the tumor “resistant” if it consists of more resistant cells than sensitive ones. Note that even if increasing the chemotherapy dose decreases the tumor volume in a short term, it may cause an increase in the resistant cell subpopulation and contribute to a switch to resistance. This leads to the subsequent iterations of therapy being inefficient (see Fig. 8). On the other hand, anti-angiogenic therapy is considered to be “resistant to drug resistance”, and thus, we assume that a small constant supply of anti-angiogenic agent is applied during the therapy. In the context of optimal treatment, it would be ideal to find a good balance between the two conflicting objectives: minimize the tumor’s volume and ensure that no switch to the resistant phenotype occurs.

In this subsection, we present results of theoretical optimization of the treatment using optimal control and our model which is based on the Hahnfeldt et al.’s model (cf. Methods for details related to the optimal control problem). First, we assume that there is a prescribed treatment that lasts assumed time consisting of constant anti-angiogenic agent supply combined with chemotherapy which is applied according to the optimal control leading to both minimizing the whole tumor size as well as preventing the tumor from becoming resistant. Nominal parameter values for which calculations have been performed are summarized in Tables 1 and 2. Treatment doses are counted as a percentage of MTD.

**Fig. 6:**
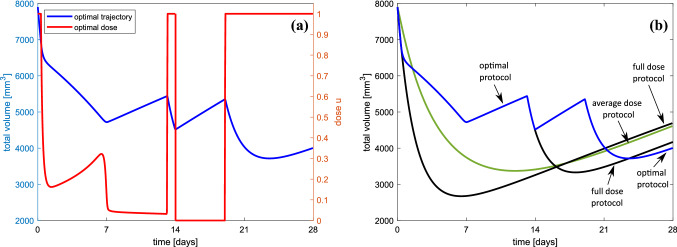
**a** Optimal protocol for 28-day treatment consisting of two 14-day therapy windows. Blue curve represents trajectory for optimal protocol penalizing drug resistance in both 14-day treatment windows. Red curve reflects the corresponding optimal dosage. **b** A comparison of the optimal trajectory (blue) with trajectories for MTD protocol (black), mean dose, which is equal to the average dose for the optimal control (green) and trajectory corresponding to 14-day optimization against drug resistance and 14-day MTD protocol (blue–black). For each protocol, initial condition is $[5900,2000,10{,}000]\ \mathrm{{mm}}^3$, just below critical volume

Figure 6a shows tumor response for two 14-day therapeutic windows. Blue curve depicts the total tumor volume during therapeutic schedules based on our optimization goal, which focuses on preventing drug resistance. Red one is a corresponding optimal dosage. The same trajectory compared to three other trajectories is depicted in Fig. 6b. The black curve represents a therapeutic schedule based on MTD protocol, which is a result of engaging the objective functional that minimizes the tumor volume during and at the end of therapy. The blue–black curve depicts the protocol that is based on preventing drug resistance during first 14 days and for the next 14 days applies MTD protocol, and the green curve reflects the average-optimal dose during 28 days. One can see that the total tumor volume at the end of the first part of therapy (day 14) is the smallest when the MTD protocol is applied, as the initially drug-sensitive tumor responses very good for treatment. Although in the middle of the therapy, the tumor is the smallest one, it is almost entirely composed of cells resistant to treatment (here, it consists of 99.72% drug-resistant cells), and thus, the repeated therapy has no effects and tumor at the end of therapy is finally the greatest (here, the tumor is almost 20% bigger than in optimal scenario). In the optimal therapeutic schedule, the drug dose is applied both as the MTD dose and singular, which prevent the tumor-resistant cell domination and results that the tumor response for treatment is better. Note that, in optimal scenario, both MTD and singular protocols are applied, but the mean dose during the first 14 days is 21.45% of MTD and during the whole 28-day therapy is 42.69%. For singular-MTD scheme, the mean dose is 60.72% of MTD.

**Fig. 7:**
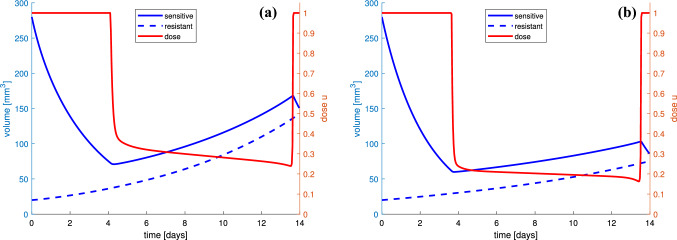
Sample solution to our control problem and corresponding optimal chemotherapy dose *u* for anti-angiogenic doses: **a** $v = 0.5$ and **b** $v=1$. In **a**, the average dose is 52.51% of MTD, while the average singular dose is 29.64% of MTD, whereas in **b**, they are 43.90% and 19.96%, respectively

In Fig. 7, we present results of optimization for nominal parameter values with $v=0.5$ and $v=1$. We see that the optimal drug dosage starts and ends with MTD; however, during around two-thirds of the whole treatment time much less doses are applied, not exceeding 30% of MTD for $v=0.5$ and 20% of MTD for $v=1$. It seems obvious that the treatment should start with MTD to decrease the whole tumor size. Then, much smaller doses could be applied to control the growth of both subpopulations and to prevent the tumor to become resistant (see Fig. 9). However, at the end, when the whole tumor size is again large, MTD is applied again. This is the result one could obtain for one cycle of the therapy.

**Fig. 8:**
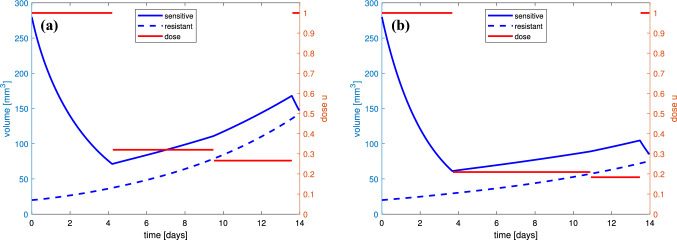
Suboptimal protocols corresponding to numerically optimal depicted in **a** Fig. 7a and **b** Fig. 7b, which consist of partially constant treatment. In both examples, a constant dosage protocol with rates given by the average-optimal control is an excellent suboptimal protocol as the suboptimal solution comes exceptionally close to the numerically optimal value—it lies within 0.2%

It should be noted that the proposed optimal treatment has a part during which the chemotherapy dose changes continuously in time. Such protocols are of course impossible to realize in practice. Therefore, following the ideas presented in Ledzewicz and Schättler (2008), we propose suboptimal protocols which consist of partially constant treatment. Figure 8 presents examples of suboptimal solutions corresponding to numerically optimal ones depicted in Fig. 7, where time-dependent controls *u* are approximated by piecewise-constant functions. In both examples, suboptimal protocol consist of, after initial full dose, two mean doses and full dose again at the end. In Fig. 8a, where anti-angiogenic dose is $v=0.5$, switching times are: $t_1=4.24$ days, $t_2 = 9.49$ days and $t_3 = 13.59$ days. The average dose is 52.72% of MTD and is 0.4% larger comparing to the optimal scenario, and both the suboptimal solution and final resistance are close to numerically optimal, lying within ±0.2%. For $v=1$ (see Fig. 8b), switching times are: $t_1=3.68$ days, $t_2=10.86$ days and $t_3=13.48$ days, and the suboptimal trajectory and final resistance are just as close to the corresponding optimal solution.

**Fig. 9:**
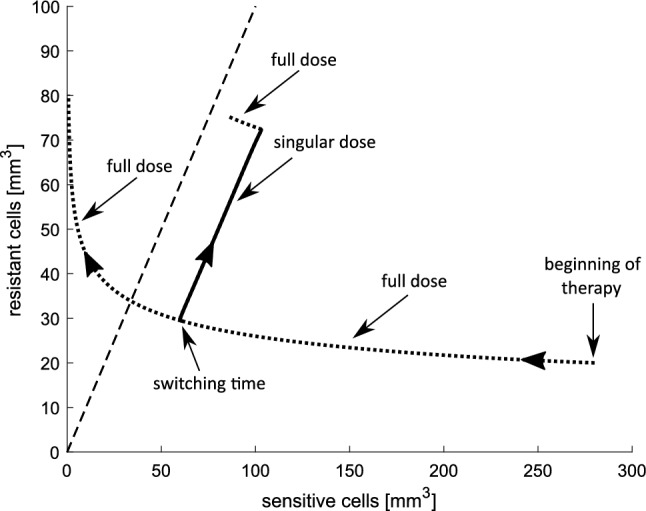
Resistant cells plotted against sensitive cells. The dashed line reflects the diagonal where numbers of sensitive and resistant cells are equal. Dotted lines depict MTD doses, while solid line represents singular dose. Initial condition is $[280,20,650]\ \mathrm{{mm}}^3$

In Fig. 9, resistant cells are plotted against sensitive ones for two protocols. Dotted lines represent MTD parts of control, while solid line depicts singular part. For this particular example, initially, the optimal control is given at full dosage until the singular curve is reached (at $t_1=3.68$ days). Then, the administration follows the time-varying singular control in one scenario, or remains MTD in the latter. The effect of preventing against drug resistance is than clearly visible. Singular control keeps the trajectories below the diagonal, while for the MTD, the tumor becomes almost completely resistant. Finally, optimal control is again realized along the trajectory for full dose, which results in the maximum tumor reduction.

We also consider possible changes of the therapeutic schemes on the parameters reflecting mutation rates. As the values of mutation rates are hard or impossible to determine, or the degree of uncertainty regarding the estimation is high, we drew 400 different random pairs of these parameters for two values of the anti-angiogenic agent dose $v=0.5$ and $v=1$, and for two different pairs of chemotherapy sensitivity parameters: $\beta _1=0.3$, $\beta =0.1$ and $\beta _1=0.6$, $\beta =0.2$. This was to assess how the results may change depending on how strongly the anti-angiogenic therapy and chemotherapy are able to suppress vasculature and tumor growth.

**Fig. 10:**
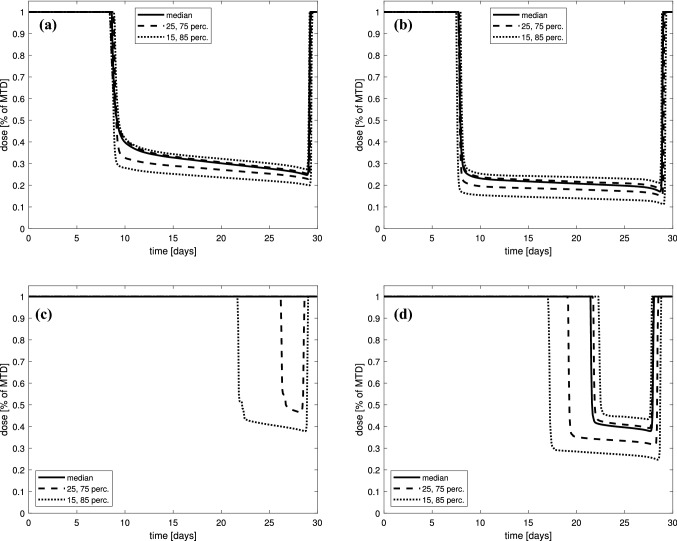
Selected percentiles of optimal dose $u_{opt}$. Mutation parameters $(\tau _1,\tau _2)$ are randomized with different values of anti-angiogenic agent doses and chemotherapy sensitivity parameters: **a** $\beta _1=0.60$, $\beta =0.20$, $v=0.5$; **b** $\beta _1=0.60$, $\beta =0.20$, $v=1$; **c** $\beta _1=0.30$, $\beta =0.10$, $v=0.5$; **d** $\beta _1=0.30$, $\beta =0.10$, $v=1$

**Fig. 11:**
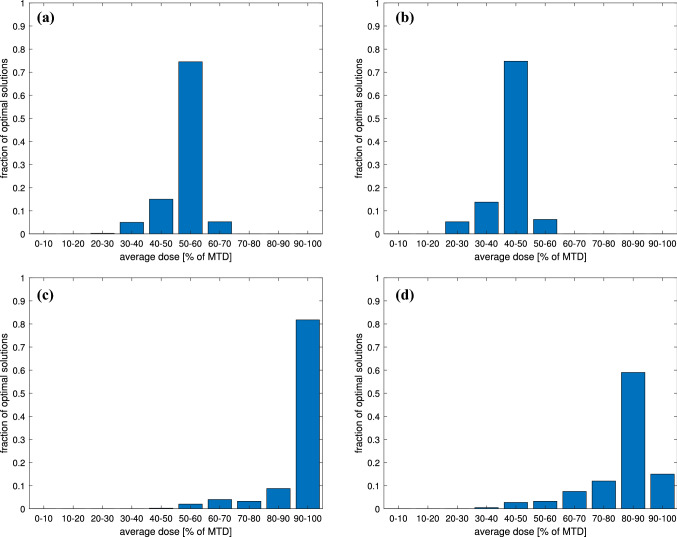
Mean dose distribution for optimal dose $u_{opt}$. Mutation parameters $(\tau _1,\tau _2)$ are randomized with different values of anti-angiogenic agent doses and chemotherapy sensitivity parameters: **a** $\beta _1=0.60$, $\beta =0.20$, $v=0.5$; **b** $\beta _1=0.60$, $\beta =0.20$, $v=1$; **c** $\beta _1=0.30$, $\beta =0.10$, $v=0.5$; **d** $\beta _1=0.30$, $\beta =0.10$, $v=1$

For a fixed time $t\in [0,T]$, we may view $u_{opt}$ as a random variable defined on the set $\{(\tau _1,\tau _2):\ \tau _1,\tau _2\in [0.00001,0.05]\}$. Figure 10 shows different percentiles of the distribution of $u_{opt}$ along the interval [0, *T*], while Figure 11 shows the distribution of the average dose. One can see that although the shape of the curve reflecting the optimal dosage is always the same, however, the portion of MTD applied differs for different mutation parameters.

Based on our numerical results, one can see that two kinds of optimal solutions are possible. Either $u_{opt}$ consists of a full-dose interval, followed by an intermediate singular dose and a very short full-dose interval at the end, or it was optimal to apply full-dose throughout the entirety of the treatment window.

**Fig. 12:**
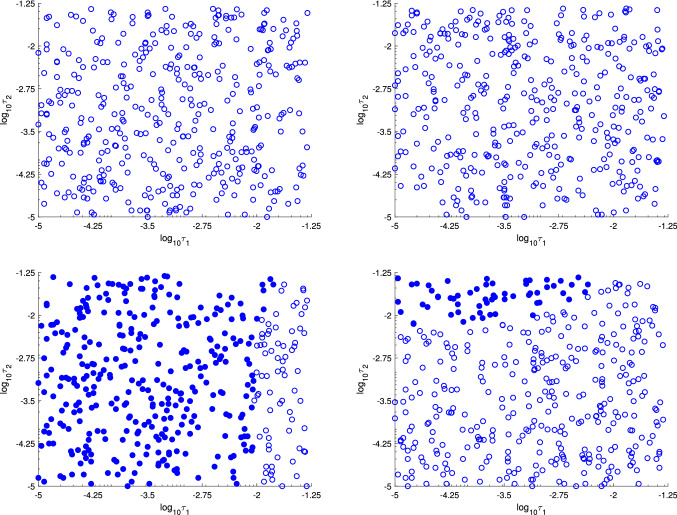
Classification of mean drug dose in optimal treatment depending on randomized mutation rates projected on the plane. Full-dose and intermediate-dose protocols are labeled by filled and empty circles, respectively. Results are shown for different pairs of chemotherapy sensitivity parameters and ani-angiogenic agent dose: **a** $\beta _1=0.60$, $\beta =0.20$, $v=0.5$; **b** $\beta _1=0.60$, $\beta =0.20$, $v=1$; **c** $\beta _1=0.30$, $\beta =0.10$, $v=0.5$; **d** $\beta _1=0.30$, $\beta =0.10$, $v=1$

As visible in Figs. 11a and 11b, when the chemotherapy sensitivity parameters are large enough, the average-optimal dosage is always intermediate and almost never exceeds 40–50% of MTD. When the chemotherapy sensitivity parameters are smaller, the average dose is larger the smaller is the anti-angiogenic agent dose, see Figs. 11c and 11d. Nonetheless, on the $(\log _{10}\tau _1,\log _{10}\tau _2)$-plane, it can be noticed that there is a clearly outlined region for which an intermediate dose is optimal (see Fig. 12c and 12d).

To identify whether a distinction between the full-dose and intermediate-dose protocols can be made in the parameter space, we classified the protocols into the two categories defined above. We consider a protocol as full-dose protocol when the average dose exceeds 90% of MTD. The rest we classify as intermediate-dose protocols. Figure 12 shows the classified protocols projected on two-dimensional parameters subspace $(\tau _1,\tau _2)$, for different anti-angiogenic agent dosages and chemotherapy sensitivity parameters. For relatively large values of sensitivity parameters, the mutation rates have no effect whether the optimal dose is full or intermediate, but for smaller values, however, it can be seen that small values of $\tau _1$ result in full-dose protocols being optimal.

## Discussion

In this study, we presented the results from a mathematical model of tumor growth under angiogenic signaling. The model tracks the number of chemotherapy-sensitive cells, the number of chemotherapy-resistant cells, and a variable carrying capacity related to the size of the vasculature. We use the proposed model to support the hypothesis that smaller doses of chemotherapy could lead to better results and to maximization of patient survival.

We based our analysis on the results and parameters obtained in Hahnfeldt et al. (1999). There is, however, a lot of uncertainty regarding the nominal values of parameters that are hard or impossible to estimate, such as mutation rates, chemotherapy sensitivity parameters, as well as the precise effects of the anti-angiogenic treatment. To examine how the solution depends on these parameters, we have performed a sensitivity analysis with respect to those parameters.

In Sect. 3.1, the therapeutic objective is to maximize patient survival time, which is defined as a time needed for the tumor to reach a critical level. As a first and simplest approximation of therapeutic scheduling, we consider scenario in which we are able to apply constant doses of both anti-angiogenic and chemotherapy for a long time. This theoretical analysis shows that, in long-term scenario, intermediate chemotherapy doses lead to the longest survival time. In Fig. 3, the survival time is plotted against different chemotherapy doses. Based on the evolution of the volumes of sensitive and resistant subpopulations for three different chemotherapy doses: smaller than optimal, optimal and larger than optimal, we have checked that in the optimal scenario, both types of tumor cells are present in a substantial numbers. The optimal dosage, which is of about 20% of MTD, maintains the tumor volume below the fatal for 120 days. In both remaining scenarios, the survival time is shorter. When the dose is too small, it is not able to effectively inhibit the tumor growth even if the tumor is still sensitive to the therapy, while for too large dose, a quick outburst of resistant subpopulation is present. Note that these simulations were performed for a fixed value of anti-angiogenic agent dose. To further investigate the model dynamics for different combinations of these two doses, we have carried out a set of simulations for different values of chemotherapy and anti-angiogenic agent doses. It is obvious that the higher the anti-angiogenic agent dose is, the smaller the vasculature size becomes, and as a result, the smaller the total tumor volume is. As shown in Fig. 4, there is a specific combination of these doses that prolongs the survival time the most. It should be noted that even a slight over-optimal dose of the drug may significantly shorten the patient survival. When optimal drug administration is applied, the tumor never reaches the critical level, which corresponds to successful treatment. Furthermore, our simulations show that low values of “toward resistance” mutation rate $\tau _1$ correspond to longer survival time and lower chemotherapy doses to be applied, see Fig. 5. It also appears that mutation toward resistance plays more important role that the opposite direction mutation $\tau _2$, as the survival time and optimal dose are far less dependent on the latter.

The standard therapeutic goal is to minimize tumor volume together with imposing some constraints on the drug dosage. In Sect. 3.2, we optimize the therapy and dose scheduling, where apart from the usual goal, the aim is also to penalize drug resistance. We call a tumor-resistant if it consists of more resistant cells than sensitive ones. Such tumors do not respond properly to treatment, which may result in therapy failure. That is why, we define a new objective to target the issue of drug resistance which penalizes resistant populations. More details can be found in Sect. 2.2. Minimizing tumor volume and ensuring that no switch to the resistant phenotype occurs become our therapeutic goal. In Fig. 6, we have compared the result of two 14-day optimal treatment schedules based on our approach with an approach based on an MTD protocol. Although the tumor initially responded very well to MTD treatment and in the middle of the therapy was the smallest, it consisted almost entirely of drug-resistant cells. This caused the regrowth to be drug resistant, which caused that further administration of the chemotherapeutic drug had no effect, and finally, at the end of therapy, the tumor was almost 20% bigger than in our optimal protocol which takes into account drug resistance. The results obtained based on average constant optimal dose are equal to 42.69% of MTD and gave a very slightly better result. The best result was obtained when intermediate, time-dependent dose was administrated during the whole course of treatment. Ultimately, tumor response to treatment was improved because of preventing the domination of resistant cells over sensitive ones.

The presented comparison was the motivation to make more detailed insight into the tumor growth dynamics and its response to treatment based on our therapeutic goal. Figure 7 shows the therapy optimization in a 14-day therapeutic window. We investigated how the therapy may be used to delay the onset of drug resistance for two values of anti-angiogenic agent doses. We showed that the optimal dosage administration should start with MTD dose, but during around 2/3 of the whole treatment time, the administrated dose should be much less, not exceeding 20%–30% of MTD. As the simulations show only one cycle of the therapy, the optimal administrating should also end with MTD dose. Nonetheless, the average dose during the whole therapy, including MTD parts, is intermediate and amounts to about 40%–50% of MTD. The impact of the singular dose administrating on the tumor response to the treatment is clearly visible in Fig. 9. The optimal dose is full dose until it reaches the singular curve. Then, the dosage should follow the time-varying singular dose, which prevents the tumor to become more resistant than sensitive to the therapy. When the dose does not switch to singular part, the tumor reaches the state in which drug-resistant cells dominate.

As the numerically optimal treatment protocols consisting time-varying schemes may not be practically applicable, we proposed suboptimal protocols consisting of partially constant treatment. Approximating the optimal control by piecewise-constant functions, we proposed protocols that consist of several mean doses, with exact switching times, which come exceptionally close to the numerically optimal values.

We have also solved the optimal control problem numerically for 400 random values of mutation parameters $\tau _1$ and $\tau _2$ with different assumptions about the chemotherapy strength (parameter $\beta _1$) and its effect on tumor vasculature ($\beta$), as well as different assumptions regarding the anti-angiogenic agent doses. In that way, we were able to check how the solution may change with changes of parameters, which numerical values are hard to be determined. We have obtained that for larger values of $\beta _1$ typically, after an initial full-dose interval, the optimal control consists of a long period of intermediate dose ranging between 20% and 40% of MTD, depending on the anti-angiogenic agent dose, as shown in Figs. 10 and 11. We have also shown that the choice between a full-dose and intermediate-dose protocols is highly dependent on mutation rates, in particular when the tumor is less sensitive to the therapy. In such case, in general, full-dose protocols are favored to be optimal. For more sensitive tumors, the intermediate-dose protocols are optimal, regardless of the anti-angiogenic agent dose; see Fig. 12a–d. This may be important in the design of protocols for more and less aggressive tumors.

## Conclusions

In this paper we proposed an extension of the Hahnfeldt et al.’s model for heterogeneous tumors. We have proposed a modified goal for therapy optimization. In addition to penalizing the tumor volume during and at the end of treatment and minimizing the dose amount, we penalize the resistance of tumor cells to the therapy. Our study showed that penalizing tumor resistance gives better results and leads to intermediate-dose protocols. Our theoretical analysis resulted in singular controls, that start and end with full dose administration and relatively small doses in between. We have shown that such intermediate doses do not exceed 20%–30% of MTD.

Optimal dosages are time-varying, and in fact, they may not be practically realizable, as they require information generally not available in continuous time. However, they can still play an important role in designing practical protocols, serving as a benchmark for other implementable protocols. In the paper, we have proposed suboptimal protocols, which are based on these numerically calculated optimal values. By comparing with optimal protocols, we can design piecewise-constant intermediate protocols and precisely indicate switching points. Suboptimal protocols described in this study are actually very close to the optimal, giving the final results laying within ± 0.2%. Furthermore, theoretical results provide a tool that can be used to design even simpler protocols based on the average-optimal dose protocols. One can note that it would be extremely difficult to derive such good suboptimal protocols without theoretical analysis of the problem. Their implementation is simple; however, it still may cause the treatment gives better results.

It should be pointed out that the optimal protocol depends on few issues, like the initial tumor volume, the sensitivity of the tumor to the treatment, and aggressiveness of the tumor, and it was observed that in general the average-optimal dose was significantly less than MTD. Sensitivity analysis showed that the choice weather the full dose or the intermediate dose was the most dependent on the value of toward resistance mutation rate, which in turn is directly related to the aggressiveness of the tumor. Lower tumor sensitivity may lead to longer MTD interval at the beginning of therapy.

Our simulations were generally limited to one cycle of therapy. It is clear that in the absence of further treatment, tumor follows the uncontrolled dynamics and starts to increase. It is also obvious that repeated administration of the drugs is necessary if one wants to control and maintain the tumor below a certain level in a long horizon of time.

The presented problem is preliminary in the sense that our model contains the most fundamental processes like mutations and sensitivity to the therapy. Our next goal is to extend the model to provide further insight into the mechanisms of action of metronomic therapy by including the response of the immune system. However, it leads to more complicated mathematical models.

## Figures and Tables

**Fig. 13:**
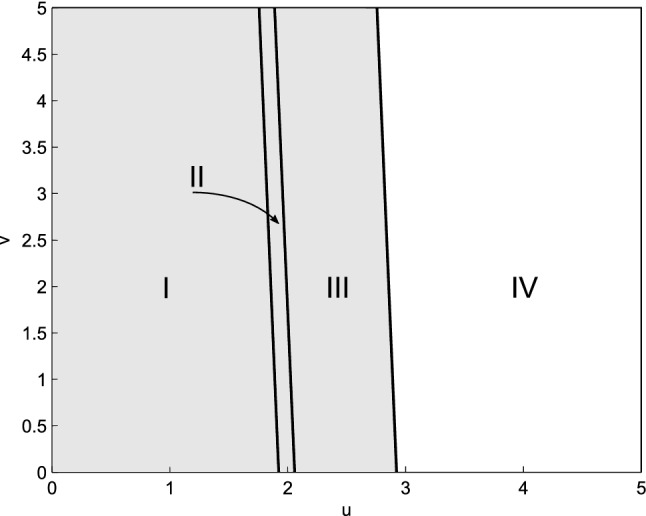
Bifurcation diagram for system (1). The gray area indicates the range of parameters for which the positive stationary state exists (regions I, II, and III), while for parameters from region IV, it does not exist. The steady state is locally stable in regions I and II, whereas in region III, it is unstable. Region I depicts the range of parameters for which the sufficient condition holds. Here, $d=2$, while the values of the remaining parameters are listed in Table 1

## Appendix: Mathematical properties of the model

### Basic properties

Due to the realistic context of our study, we are interested in the existence and non-negativity of solutions of System (1). Let us now assume that *u* and *v* are bounded and at least continuous functions.

Let us define set $\Omega =({\mathbb {R}}^+)^3$. In the absence of the treatment, i.e., when $u=v\equiv 0$, the right-hand side of System (1) is of class $C^{\infty }$, and in the presence of the treatment, it is of the same class as the functions *u* and *v*, i.e., at least continuous with respect to time which guarantees local existence and uniqueness of solutions with a given initial condition.

#### Proposition 1

The set $\Omega$ is positively invariant under System (1).

#### Proof

At first, note that $N_1=N_2=0$ is not admissible. If $N_1=0$, then ${\dot{N}}_1 = \tau _2N_2$, and for $N_1$ to be negative, it is required that $N_2\le 0$. Similarly, if $N_2=0$, then $N_2$ becomes negative if and only if $N_1$ becomes negative first. Thus, starting with non-negative initial condition, where not both $N_1$ and $N_2$ equal zero, then both $N_1$ and $N_2$ remain non-negative.

Assume that $K({\bar{t}})=0$ at some time moment ${\bar{t}}>0$. Then, ${\dot{K}}({\bar{t}})=b\left( N_1({\bar{t}})+N_2({\bar{t}})\right) >0$, so *K* cannot become negative. $\square$

#### Proposition 2

For initial condition $(N_1(0),N_2(0),K(0))\in \Omega$, the solution of System (1) exists for all $t\ge 0$.

#### Proof

By the non-negativity of $N_1$ and $N_2$ and positivity of parameters, using the inequality $-\ln x\le \frac{1}{x}-1$, we can estimate

$$\begin{aligned} \begin{aligned} {\dot{N}}_i&\le \lambda _iN_i\left( \frac{K}{N_1+N_2}-1\right) +\tau _jN_j \\&\le \lambda _iK\frac{N_i}{N_1+N_2} + \tau _jN_j \\&\le \lambda _iK+\tau _jN_j, \\ {\dot{K}}&\le b(N_1+N_2), \end{aligned} \end{aligned}$$

for $i,j=1,2$, $i\not =j$. It follows:

$$\begin{aligned} \frac{d}{dt}\left( N_1+N_2+K\right) \le c\left( N_1+N_2+K\right) , \end{aligned}$$

where $$c = \max \{\lambda _1+\lambda _2, \tau _1+b,\tau _2+b\}$$. Thus

$$\begin{aligned} N_1+N_2+K\le O\left( \mathrm {e}^{ct}\right) \end{aligned}$$

for $t\ge 0$ and the solution exists for all $t\ge 0$. $\square$

### Analysis of the model for constant drug dosage

As a first and simplest approximation to the model dynamics, we consider constant dosage of both drugs, $u(t) \equiv u$, $v(t) \equiv v$.

#### Steady states

##### Proposition 3

System (1) has only one positive steady $S^*=({\bar{N}}_1,{\bar{N}}_2,{\bar{K}})$ with coordinates defined by

2 $$\begin{aligned} {\bar{N}}_1 = {\bar{K}}\mathrm {e}^\alpha \frac{2A}{2A-B+\sqrt{\Delta }},\ {\bar{N}}_2 = {\bar{K}}\mathrm {e}^\alpha \frac{-B+\sqrt{\Delta }}{2A-B+\sqrt{\Delta }}, \ {\bar{K}} = \frac{\left( b\mathrm {e}^\alpha -\mu -\beta u -\gamma v\right) ^{3/2}}{d^{3/2}\mathrm {e}^\alpha }, \end{aligned}$$

which exists only if

3 $$\begin{aligned} b\mathrm {e}^\alpha -\mu -\beta u-\gamma v>0, \end{aligned}$$

where $\alpha$ is defined by

4 $$\begin{aligned} \begin{aligned} \alpha =\frac{ \frac{\sqrt{\Delta }-B}{2A}\tau _1-\tau _2}{\lambda _2}, \quad \Delta&=B^2-4AC = \left( \lambda _2\tau _1-\lambda _1\tau _2+\beta _1\lambda _2u\right) ^2+4\lambda _1\lambda _2\tau _1\tau _2, \\ A&=\lambda _1\tau _1,\quad B=\lambda _2\tau _1-\lambda _1\tau _2+\beta _1\lambda _2u, \quad C=-\lambda _2\tau _2. \end{aligned} \end{aligned}$$

##### Proof

We first examine the existence of steady states of System (1). Note that there are no semi-positive steady states as well as (0, 0, 0) is not an admissible point. Adding the first two equations, we obtain

$$\begin{aligned} \ln \left( \frac{N_1+N_2}{K}\right) =-\frac{\beta _1N_1u}{\lambda _1N_1+\lambda _2N_2}, \end{aligned}$$

and substituting it to the second equation, we have

$$\begin{aligned} \beta _1\lambda _2uN_1N_2+\left( \lambda _1N_1+\lambda _2N_2\right) \left( \tau _1N_1-\tau _2N_2\right) =0. \end{aligned}$$

At a steady state, we have both $N_1>0$ and $N_2>0$. Thus, dividing the above equation by $N_2^2$, we obtain

$$\begin{aligned} \lambda _1\tau _1\left( \frac{N_1}{N_2}\right) ^2 + \left( \lambda _2\tau _1-\lambda _1\tau _2+\beta _1\lambda _2u\right) \frac{N_1}{N_2} -\lambda _2\tau _2=0. \end{aligned}$$

Defining a new variable $x=\frac{N_1}{N_2}$, we finally obtain a quadratic equation in the variable *x*

$$\begin{aligned} Ax^2+Bx+C=0, \end{aligned}$$

where *A*, *B*, and *C* are defined in (4), with positive discriminant $\Delta >0$. Using Vieta’s formula

$$\begin{aligned} x_1\cdot x_2=-\frac{\lambda _2\tau _2}{\lambda _1\tau _1}<0, \end{aligned}$$

we know that the quadratic equation has one positive and one negative root. By the definition of the variable *x*, we have

5 $$\begin{aligned} \frac{N_1}{N_2}=R, \quad R=\frac{\sqrt{\Delta }-B}{2A}>0. \end{aligned}$$

Substituting it to the second equation of (1), we obtain

6 $$\begin{aligned} N_1+N_2 = K \mathrm {e}^\alpha , \end{aligned}$$

where $\alpha$ is defined by (4). By determining $N_1=N_2\tfrac{\sqrt{\Delta }-B}{2A}$ and substituting it to (6), we get

$$\begin{aligned} N_1 = K\mathrm {e}^\alpha \frac{2A}{2A-B+\sqrt{\Delta }}>0, \quad N_2 = K\mathrm {e}^\alpha \frac{\sqrt{\Delta }-B}{2A-B+\sqrt{\Delta }}>0. \end{aligned}$$

Finally, from the third equation of (1), we calculate

$$\begin{aligned} K=\frac{\left( b\mathrm {e}^\alpha -\mu -\beta u -\gamma v\right) ^{3/2}}{d^{3/2}\mathrm {e}^\alpha }, \end{aligned}$$

which exists only if $b\mathrm {e}^\alpha -\mu -\beta u-\gamma v>0$. Eventually, we obtain that System (1) has only one positive steady state $S^*$ with coordinates satisfying (2), existing providing that (3) is satisfied. $\square$

Let us recall that for parameters estimated by Hahnfeldt et al. (1999), there is $\mu \approx 0$ which means that $S^*$ always exists for sufficiently small *u* and *v*.

#### Stability analysis

##### Proposition 4

Let us now assume that the positive steady state of System (1) exists. Then, it is locally stable if

7 $$\begin{aligned} b \left( {{\bar{K}}} \mathrm {e}^{\alpha }-1\right) +\frac{2}{3}d\root 3 \of {{\bar{K}}^2\mathrm {e}^{-\alpha }} >0,\quad {{\bar{K}}}=\frac{\left( b\mathrm {e}^\alpha -\mu -\beta u -\gamma v\right) ^{3/2}}{d^{3/2}\mathrm {e}^\alpha }, \end{aligned}$$

and unstable for the inverse inequality.

##### Proof

The Jacobian matrix of System (1) at a point $S=(N_1,N_2,K)$ reads

$$\begin{aligned}&J(S) = \\&\quad \left( \begin{array}{ccc} -\lambda _1\ln \left( \frac{N_1+N_2}{K}\right) -\lambda _1\frac{N_1}{N_1+N_2}-\tau _1-\beta _1u &{} -\lambda _1\frac{N_1}{N_1+N_2} + \tau _2 &{} \lambda _1\frac{N_1}{N_1+N_2}\cdot \frac{1}{K} \\ -\lambda _2\frac{N_2}{N_1+N_2}+\tau _1 &{}-\lambda _2\ln \left( \frac{N_1+N_2}{K}\right) -\lambda _2\frac{N_2}{N_1+N_2}-\tau _2 &{} \lambda _2\frac{N_2}{N_1+N_2}\cdot \frac{1}{K} \\ b-\frac{2}{3}dK\left( N_1+N_2\right) ^{-1/3} &{} b-\frac{2}{3}dK\left( N_1+N_2\right) ^{-1/3} &{} -\mu -d\left( N_1+N_2\right) ^{2/3}-\beta u - \gamma v \end{array}\right) . \end{aligned}$$

From the first equation of System (1), at a steady state, we have

$$\begin{aligned} -\lambda _1\ln \left( \frac{N_1+N_2}{K}\right) -\lambda _1\frac{N_1}{N_1+N_2}-\tau _1-\beta _1u = -\lambda _1\frac{N_1}{N_1+N_2}- \tau _2\frac{N_2}{N_1}, \end{aligned}$$

and similarly from the second equation

$$\begin{aligned} -\lambda _2\ln \left( \frac{N_1+N_2}{K}\right) -\lambda _2\frac{N_2}{N_1+N_2}-\tau _2 = -\lambda _2\frac{N_2}{N_1+N_2}-\tau _1\frac{N_1}{N_2}, \end{aligned}$$

while from the third equation

$$\begin{aligned} -\mu -d\left( N_1+N_2\right) ^{2/3}-\beta u - \gamma v = -b\frac{N_1+N_2}{K}. \end{aligned}$$

By the relations above and using (5) and (6), we obtain

$$\begin{aligned} \lambda _1\frac{{{\bar{N}}}_1}{{{\bar{N}}}_1+{{\bar{N}}}_2}+ \tau _2\frac{\bar{N}_2}{{{\bar{N}}}_1} = \frac{\lambda _1R}{1+R}+\frac{\tau _2}{R}, \ \lambda _2\frac{{{\bar{N}}}_2}{{{\bar{N}}}_1+{{\bar{N}}}_2}+\tau _1\frac{\bar{N}_1}{{{\bar{N}}}_2} = \frac{\lambda _2}{1+R} + \tau _1 R, \ b\frac{\bar{N}_1+{{\bar{N}}}_2}{K}= b\mathrm {e}^{\alpha }, \end{aligned}$$

and therefore at the steady state $S^*$

$$\begin{aligned} J(S^*) = \left( \begin{array}{ccc} -\frac{\lambda _1R}{1+R}- \frac{\tau _2}{R} &{} -\frac{\lambda _1R}{1+R} + \tau _2 &{} \frac{\lambda _1R}{1+R}\cdot \frac{1}{{\bar{K}}} \\ -\frac{\lambda _2}{1+R}+\tau _1 &{} -\frac{\lambda _2}{1+R}-\tau _1R &{} \frac{\lambda _2}{1+R}\cdot \frac{1}{{\bar{K}}} \\ b-\frac{2}{3}d\root 3 \of {{\bar{K}}^2\mathrm {e}^{-\alpha }} &{} b-\frac{2}{3}d\root 3 \of {{\bar{K}}^2\mathrm {e}^{-\alpha }} &{} -b\mathrm {e}^{\alpha } \end{array}\right) ; \end{aligned}$$

hence, the characteristic polynomial reads

$$\begin{aligned} {\mathcal {P}}(\lambda )=\lambda ^3+a_2\lambda ^2+a_1\lambda +a_0, \end{aligned}$$

where

$$\begin{aligned} \begin{aligned} a_2 =&\ \frac{\lambda _1R}{1+R}+\frac{\lambda _2}{1+R} + \tau _1R +\frac{\tau _2}{R} + b\mathrm {e}^{\alpha }, \\ a_1 =&\ \frac{1}{{\bar{K}}}\left( b\bar{K}\mathrm {e}^{\alpha }-b+\frac{2}{3}d \root 3 \of {{\bar{K}}^2\mathrm {e}^{-\alpha }}\right) \cdot \left( \frac{\lambda _1R}{1+R}+\frac{\lambda _2}{1+R}\right) + \left( \lambda _1\tau _1R+\frac{\lambda _2\tau _2}{R} \right) \\&+ \left( \tau _1R+\frac{\tau _2}{R}\right) b\mathrm {e}^{\alpha }, \\ a_0 =&\ \frac{1}{{\bar{K}}}\left( b\bar{K}\mathrm {e}^{\alpha }-b+\frac{2}{3}d \root 3 \of {{\bar{K}}^2\mathrm {e}^{-\alpha }}\right) \cdot \left( \lambda _1\tau _1R+\frac{\lambda _2\tau _2}{R}\right) . \end{aligned} \end{aligned}$$

By the Routh–Hurwitz criterion, the steady state $S^*$ is locally stable if $a_2$, $a_1$, $a_0>0$, and $a_1a_2>a_0$. It is obvious that $a_2>0$. The condition $a_0>0$ holds only if (7) holds. Note that under this assumption, we have also $a_1>0$. Moreover, it is obvious that if $a_0<0$ (i.e., the inequality inverse to (7) holds), then $S^*$ is unstable.

From the third equation of System (1), at the steady state, we can estimate

$$\begin{aligned} b\mathrm {e}^{\alpha } = \mu + d \left( {\bar{K}}\mathrm {e}^{\alpha }\right) ^{2/3} + \beta u + \gamma v > \frac{2}{3} d \left( {\bar{K}}\mathrm {e}^{\alpha }\right) ^{2/3}, \end{aligned}$$

implying

$$\begin{aligned} b > \frac{2}{3}d \root 3 \of {{\bar{K}}^2\mathrm {e}^{-\alpha }} . \end{aligned}$$

Now, assuming (7), note that multiplying the last term from $a_2$ by the second term from $a_1$, we obtain

$$\begin{aligned}&a_1a_2> b\mathrm {e}^{\alpha } \left( \lambda _1\tau _1R+\frac{\lambda _2\tau _2}{R} \right) \\&\quad >b\mathrm {e}^{\alpha } \left( \lambda _1\tau _1R+\frac{\lambda _2\tau _2}{R} \right) - \left( b - \frac{2}{3}d \root 3 \of {{\bar{K}}^2\mathrm {e}^{-\alpha }}\right) \left( \lambda _1\tau _1R+\frac{\lambda _2\tau _2}{R} \right) = a_1. \end{aligned}$$

$$\square$$

##### Proposition 5

If

8 $$\begin{aligned} b\text {e}^{\alpha }-\mu -\beta u-\gamma v >d, \end{aligned}$$

then the positive steady state $S^*$ of System (1) exists and is locally stable.

##### Proof

It is obvious that if (8) holds, then $S^*$ exists. Moreover

$$\begin{aligned} {{\bar{K}}} = \frac{\left( b\mathrm {e}^\alpha -\mu -\beta u -\gamma v\right) ^{3/2}}{d^{3/2}\mathrm {e}^\alpha }> \frac{d^{3/2}}{d^{3/2}\mathrm {e}^{\alpha }}=\mathrm {e}^{-\alpha } \ \Longrightarrow \ {{\bar{K}}} \mathrm {e}{^{\alpha }} > 1 . \end{aligned}$$

Hence, $b \left( {{\bar{K}}} \mathrm {e}{^{\alpha }} - 1\right) >0$ and (7) is then satisfied. $\square$

To sum up, we can state that condition (3) is necessary for the existence of the positive steady state and (7) is necessary for its stability, while condition (8) is sufficient for both existence and stability. As these conditions are difficult to interpret, we investigated the model dynamics numerically for fixed values of the parameters. The qualitative properties of the system are strictly determined by the values of therapy doses, and thus, we can treat drug dose *u* and anti-angiogenic agent dose *v* as bifurcation parameters.

We can check numerically that for nominal values of the parameters, the positive steady state always exists and is locally stable. Ranges of parameters *u* and *v* for which necessary and sufficient conditions hold are depicted in Fig. 13. Note that the influence of the value of the anti-angiogenic agent dose on the existence and stability of the steady state is less significant than the chemotherapy dose. To obtain a better insight into the bifurcation dynamics, simulations were performed for $d=2$. Note that the condition under which the positive steady state exists does not depend on this parameter.
